# Genetic variants in the *acylphosphatase* 2 gene and the risk of breast cancer in a Han Chinese population

**DOI:** 10.18632/oncotarget.13495

**Published:** 2016-11-22

**Authors:** Fuli Zhang, Yan Zhang, Zhiping Deng, Pengcheng Xu, Xiyang Zhang, Tianbo Jin, Qiufang Liu

**Affiliations:** ^1^ Department of Oncology Surgery, Traditional Chinese Medicine Hospital of Xi’an, Xi’an, Shaanxi 710021, China; ^2^ Department of Breast Surgery, Tumor Hospital of Shaanxi Province, Xi’an 710061, China; ^3^ Inner Mongolia Medical University, Hohhot 010010, Inner Mongolia, China; ^4^ School of Life Sciences, Northwest University, Xi’an, Shaanxi 710069, China; ^5^ Key Laboratory of Resource Biology and Biotechnology, Ministry of Education, Northwest University, Xi’an, Shaanxi 710069, China; ^6^ Department of Radiotherapy, Tumor Hospital of Shaanxi Province, Xi’an 710061, China

**Keywords:** breast cancer, ACYP2, single nucleotide polymorphism, association study

## Abstract

We performed a case-control study to investigate the associations between seven single nucleotide polymorphisms (SNPs) in the *acylphosphatase* 2 (*ACYP2*) gene and breast cancer (BC) risk in a Han Chinese population. There were 183 BC cases and 195 healthy controls included in the study. The SNPs were genotyped using the Sequenom MassARRAY platform. Logistic regression (adjusted for age group, body mass index [BMI], and menopause status), was used to evaluate the associations between the various genotypes and BC risk. Statistical analysis revealed that rs12621038 was associated with a decreased risk of BC in the allele (T vs. C: odds ratio [OR] = 0.71, 95% confidence interval [95% CI] = 0.52–0.94; *p* = 0.016), homozygous (TT vs. CC: OR = 0.47, 95% CI = 0.24–0.85; *p* = 0.014), dominant (OR = 0.62; 95% CI = 0.40−0.96; *p* = 0.032), and additive (OR = 0.68; 95% CI = 0.50–0.92; *p* = 0.012) models. In addition, we found that rs1682111 and rs17045754 were associated with the risk of BC and correlated with recurrence, and that rs6713088 correlated with tumor size. In sum, our findings reveal significant associations between SNPs in the *ACYP2* gene and BC risk in a Han Chinese population.

## INTRODUCTION

Breast cancer (BC) is a lethal malignancy that arises in the breast tissue or ducts. It is a major cause of morbidity and mortality in women worldwide [1]. There were an estimated 231,840 new cases of invasive BC among U.S. women in 2015 and 40,290 BC deaths [2]. BC risk factors include age, genetics, family history, younger age at menarche, and older age at menopause [3, 4]. Approximately 5%–10% of all BC cases are hereditary [5]. Previous studies have identified several genes associated with BC susceptibility in various populations such as *ACYP2*, *MACC1, BRCA1, BRCA2, PTEN, CHEK2, BACH1, PALB2, RAD50*, and *TP53*. [6–10]. Interestingly, rs11125529 in *ACYP2* was associated with a risk of hormone-related cancers (breast, ovarian, and prostate) in a European population, [11, 12].

The *ACYP2* gene is located on human chromosome 2 (2p16.2). This gene encodes an acylphosphatase (AcPase) that hydrolyzes the phosphoenzyme intermediates of various membrane pumps (e.g. the sarcoplasmic reticulum Ca^2+^/Mg^2+^-ATPase found in skeletal muscle) [13, 14]. It also has a potential role in maintaining Ca^2+^ homeostasis [15]. The primary functions of *ACYP2* include pyruvate metabolism [11], cell differentiation [16–18], and programmed cell death (apoptosis) [19]. Apoptosis is involved in a variety of biological processes including embryonic development, immune system regulation, tissue homeostasis, and tumor suppression. Therefore, *ACYP2* may regulate apoptosis [19, 20], and mutations in *ACYP2* may promote tumorigenesis.

Previous studies have demonstrated that overexpression of *ACYP2* promotes the differentiation of SH-SY5Y neuroblastoma cells [21]. Additionally, *ACYP2* was associated with colorectal cancer metastasis [22]. However, few studies have investigated the association between *ACYP2* and BC risk, particularly in a Chinese population. Here, we investigated whether seven SNPs in *ACYP2* (rs6713088, rs12621038, rs1682111, rs843752, rs10439478, rs17045754, and rs843720) were associated with BC risk in a Han Chinese population. Our data indicate common SNPs in *ACYP2* are associated with BC susceptibility.

## RESULTS

A total of 183 patients with BC and 195 healthy individuals were enrolled in this study. The clinicopathological characteristics of the cases and controls are shown in Table 1. The average age was 46.40 ± 9.38 years and 45.35 ± 6.90 years in the case and control populations, respectively. The menopausal status (*p* = 0.716) and body mass index (BMI) (*p* = 0.056) distributions were similar between the BC patients and healthy controls. The basic information for the analyzed SNPs (chromosomal position, gene, allele, Hardy-Weinberg Equilibrium [HWE] test results, and minor allele frequency [MAF]) are shown in Table 2. The SNPs were in agreement with HWE (*p* > 0.05). The SNP call rate threshold was 98%.

**Table 1:** Distributions of select variables in breast cancer patients and healthy controls

Characteristics		Cases	Controls	*p* value
Number		183	195	
Age (mean ± SD)		46.4 ± 9.38	45.35 ± 6.89	0.218^a^
BMI, kg/m^2^ (mean ± SD)		23.08 ± 3.00	22.53 ± 2.55	0.056^a^
Menopause status				0.716^b^
	Premenopausal	115	119	
	Postmenopausal	68	76	
Age of menarche	≤ 12	25		
	> 12	158		
Tumor size (cm)	≤ 3	94		
	> 3	89		
Breastfeeding duration (month)	≤ 6	12		
	> 6	158		
Primiparous age	< 30	170		
	≥ 30	6		
Procreative times	< 1	142		
	≥ 1	33		
Clinical stage	I/II	153		
	III/IV	48		
LN metastasis	Negative	105		
	Positive	75		
Family tumor history	Negative	156		
	Positive	27		
Incipient or recurrent	Incipient	109		
	Recurrent	73		
ER	Negative	60		
	Positive	123		
PR	Negative	75		
	Positive	108		
Tumor location	Left	84		
	Right	97		
	Both	2		
Tumor type	Infiltrating ductal carcinoma	165		
	Other	18		
Fertility status	Negative	4		
	Positive	116		

LN metastasis = lymph node metastasis; ER = estrogen receptor; PR = progesterone receptor.

BMI = body mass index.

a *p* value was calculated by *t* test.

b *p* value was calculated by Pearson’s x^2^ test.

**Table 2:** Allele frequencies in cases and controls, and odds ratio estimates for breast cancer risk

SNP ID	Gene	Chromosome position	Base change	MAF-cases	MAF-controls	HWE test *p*-value	OR	95%	CI	*p*
rs6713088	*ACYP2*	2p16.2	G/C	0.410	0.428	0.884	0.929	0.695	1.241	0.617
rs12621038	*ACYP2*	2p16.2	T/C	0.383	0.469	0.666	0.701	0.524	0.936	0.016*
rs1682111	*ACYP2*	2p16.2	A/T	0.322	0.256	1.000	1.380	1.006	1.892	0.045*
rs843752	*ACYP2*	2p16.2	G/T	0.303	0.300	0.305	1.016	0.744	1.386	0.922
rs10439478	*ACYP2*	2p16.2	C/A	0.385	0.441	0.193	0.795	0.595	1.063	0.122
rs17045754	*ACYP2*	2p16.2	C/G	0.145	0.205	0.508	0.656	0.448	0.960	0.029*
rs843720	*ACYP2*	2p16.2	G/T	0.295	0.305	0.866	0.953	0.698	1.301	0.763

MAF = minor allele frequency; HWE = Hardy-Weinberg Equilibrium.

OR= odds ratio; 95% CI = 95 % confidence interval.

* *p* ≤ 0.05 indicates statistical significance.

The genotype counts for SNPs in *ACYP2* (rs6713088, rs12621038, rs1682111, rs843752, rs10439478, rs17045754, and rs843720) are shown in Table 3. The frequency distribution of rs12621038 genotypes in BC patients was the following: CC, CT, and TT. The TT genotype was associated with a decreased risk of BC compared to the CC genotype (TT vs. CC: OR = 0.47, 95% CI = 0.24–0.85; *p* = 0.014). Additionally, the GC genotype of rs17045754 was associated with a decreased risk of BC compared to the GG genotype (GC vs. GG: OR = 0.58, 95% CI = 0.37–0.92; *p* = 0.020). Finally, the AT genotype of rs1682111 was associated with an increased risk of BC compared to the TT genotype (AT vs. TT: OR = 1.56, 95% CI = 1.01–2.39; *p* = 0.043).

**Table 3:** Genotypes among the cases and controls and the associations with breast cancer risk (adjusted for age group, body mass index, and menopause status)

SNP	Genotype	Controls	Cases	OR (95% CI)	95% CI		*p*
rs6713088	C/C	64	63				1.000
	C/G	94	90	1.00	0.63	1.58	0.993
	G/G	36	30	0.88	0.48	1.60	0.667
rs12621038	C/C	53	67				1.000
	C/T	101	92	0.68	0.43	1.08	0.105
	T/T	41	24	0.46	0.24	0.85	0.014*
rs1682111	T/T	108	81				1.000
	T/A	74	86	1.56	1.01	2.39	0.043*
	A/A	13	16	1.67	0.75	3.70	0.209
rs843752	T/T	92	67				1.000
	T/G	89	91	0.84	0.55	1.29	0.418
	G/G	14	25	1.33	0.62	2.84	0.465
rs10439478	A/A	56	67				1.000
	A/C	105	91	0.71	0.45	1.13	0.147
	C/C	33	25	0.64	0.34	1.20	0.163
rs17045754	G/G	121	135				1.000
	G/C	68	43	0.58	0.37	0.92	0.020*
	C/C	6	5	0.78	0.23	2.68	0.693
rs843720	T/T	93	90				1.000
	T/G	85	78	0.93	0.60	1.42	0.725
	G/G	17	15	0.86	0.40	1.85	0.700

CI = confidence interval; OR = odds ratio; SNP = single nucleotide polymorphism.

* *p* ≤ 0.05 indicates statistical significance.

We next analyzed the associations between SNPs in *ACYP2* and patient clinicopathological features including age of menarche, tumor size (cm), duration of breast feeding (months), primiparous age, procreative times), clinical stage, lymph node (LN) metastasis, family history of cancer, incipient or recurrent tumor, estrogen receptor (ER) status, progesterone receptor (PR) status, tumor type, and fertility status. The results for positive associations are shown in Table 4. The GC + CC genotype of rs6713088 was present at a lower frequency in the tumor size > 3 cm cases (OR = 0.47, 95% CI = 0.26–0.91; *p* = 0.02) than the CC genotype. Moreover, the GC genotype of rs17045754 was present at a higher frequency in patients with BC recurrence (OR = 2.06, 95% CI = 1.05–4.034; *p* = 0.04) than the GG + CC genotype. There were no significant differences between the other clinicopathological features in the cases and controls (*p* > 0.05).

**Table 4:** The association between *ACYP2* polymorphisms and the clinical characteristics of breast cancer patients

Variables	rs6713088					rs1682111					rs17045754				
	CC	GG + GC	*p*	OR^F^	95% CI	TT	TA + AA	*p*	OR^F^	95% CI	GG	GC + CC	*p*	OR^F^	95% CI
Age of menarche															
≤ 12	8	17	0.783	1.14	0.05–2.79	10	15	0.231	1.22	0.52–2.89	21	4	0.218	0.49	0.16–1.52
> 12	55	103		1.00	(reference)	87	71		1.00	(reference)	114	44		1.00	(reference)
Tumor size (cm)															
≤ 3	25	69		1.00	(reference)	44	50		1.00	(reference)	70	24		1.00	(reference)
> 3	38	51	0.023*	0.47	0.26–0.91	37	52	0.476	1.24	0.69–2.22	65	24	0.826	1.08	0.56–2.08
Breast feeding duration (month)															
≤ 6	1	11	0.089	6.04	0.76–48.00	6	6	0.671	0.78	0.24–2.51	10	2	0.481	0.57	0.12–2.71
> 6	56	102		1.00	(reference)	69	89		1.00	(reference)	117	41		1.00	(reference)
Primiparous age															
<30	59	111		1.00	(reference)	72	98		1.00	(reference)	128	42		1.00	(reference)
≥30	1	5	0.377	0.38	0.04–3.30	6	0	--	--	--	4	2	0.634	0.66	0.12–3.71
Procreative times															
< 1	44	98	0.059	2.10	0.97–4.53	62	80	0.852	1.08	0.50–2.30	108	34	0.449	0.72	0.41–1.67
≥ 1	16	17		1.00	(reference)	15	18		1.00	(reference)	23	10		1.00	(reference)
Clinical stage															
I/II	46	89	0.866	0.94	0.47–1.88	54	81	0.053	0.52	0.27–1.01	98	37	0.544	0.79	0.36–1.70
III/IV	17	31		1.00	(reference)	27	21		1.00	(reference)	37	11		1.00	(reference)
LN metastasis															
Negative	38	67		1.00	(reference)	49	56		1.00	(reference)	77	28		1.00	(reference)
Positive	23	52	0.441	1.28	0.68–2.41	31	44	0.478	1.24	0.64–2.26	55	20	1.000	1.00	0.51–1.95
Family tumor history															
Negative	53	103		1.00	(reference)	72	84		1.00	(reference)	116	40		1.00	(reference)
Positive	10	17	0.757	0.88	0.37–2.04	9	18	0.219	1.71	0.73–4.05	19	8	0.664	1.22	0.50–3.01
Incipient or recurrent															
Incipient	40	69		1.00	(reference)	39	70		1.00	(reference)	87	22		1.00	(reference)
Recurrent	23	50	0.471	1.26	0.67–2.36	28	45	0.724	0.90	0.49–1.65	48	25	0.035*	2.06	1.05–4.04
ER															
Negative	25	35		1.00	(reference)	29	31		1.00	(reference)	45	15		1.00	(reference)
Positive	38	85	0.151	1.60	0.84–3.03	52	71	0.439	1.28	0.69–2.37	90	33	0.791	1.10	0.54–2.23
PR															
Negative	29	46		1.00	(reference)	35	40		1.00	(reference)	55	20		1.00	(reference)
Positive	34	74	0.315	1.37	0.74–2.54	46	62	0.585	1.18	0.65–2.13	80	28	0.911	0.96	0.49–1.88
Tumor type															
Infiltrating ductal carcinoma	56	109	0.675	1.24	0.46–3.37	75	90	0.330	0.60	0.22–1.68	120	45	0.338	1.88	0.52–6.79
Other	7	11		1.00	(reference)	6	12		1.00	(reference)	15	3		1.00	(reference)
Fertility status															
Negative	3	4	0.634	0.69	0.15–3.18	3	4	0.939	1.06	0.23–4.88	3	4	0.077	4.00	0.86–18.57
Positive	60	116		1.00	(reference)	78	98		1.00	(reference)	132	44		1.00	(reference)

OR = odds ratio; 95% CI = 95 % confidence interval; LN metastasis = lymph node metastasis;

ER = estrogen receptor; PR= progesterone receptor.

* *p* ≤ 0.05 indicates statistical significance.

F Adjusted for age group, body mass index (BMI), and menopause status.

Three genetic models (dominant, recessive, and additive) were used to analyze the associations between the SNPs and BC risk. The results of logistic regression analyses are shown in Table 5. We assumed that the minor allele of each SNP was a risk allele compared to wild type. We found that the minor allele T of rs12621038 was associated with a decreased risk of BC in the dominant (OR = 0.62; 95% CI = 0.40–0.96; *p* = 0.032) and additive (OR = 0.68; 95% CI = 0.50–0.92; *p* = 0.012) models. Similar results were obtained for rs17045754. It was associated with a decreased risk of BC in both the dominant (*p* = 0.022) and additive (*p* = 0.041) models. The A allele of rs1682111 was associated with an increased risk of BC risk in the dominant (OR = 1.57; 95% CI = 1.04–2.37; *p* = 0.031) and additive (OR = 1.41; 95% CI = 1.01–1.95; *p* = 0.042) models. We performed a Bonferroni correction for multiple comparisons. However, none of the associations were statistically significant after correction.

**Table 5:** Association between SNPs in *ACYP2* and breast cancer risk in dominant, recessive, and additive models after adjusting for age group, body mass index, and menopause status

SNP	Minor allele	Dominant model				Recessive model				Additive model			
		OR	95% CI		*p*	OR	95% CI		*p*	OR	95% CI		*p*
rs6713088	G	0.96	0.63	1.49	0.870	0.88	0.51	1.50	0.633	0.95	0.70	1.27	0.710
rs12621038	T	0.62	0.40	0.96	0.032*	0.58	0.33	1.01	0.054	0.68	0.50	0.92	0.012*
rs1682111	A	1.57	1.04	2.37	0.031*	1.36	0.63	2.94	0.436	1.41	1.01	1.95	0.042*
rs843752	G	0.90	0.60	1.36	0.632	1.44	0.69	3.00	0.327	1.01	0.73	1.39	0.953
rs10439478	C	0.69	0.45	1.08	0.103	0.78	0.44	1.38	0.401	0.78	0.57	1.06	0.112
rs17045754	C	0.60	0.38	0.93	0.022*	0.92	0.27	3.13	0.895	0.67	0.45	0.98	0.041*
rs843720	G	0.92	0.61	1.38	0.671	0.89	0.43	1.87	0.763	0.93	0.67	1.28	0.643

OR = odds ratio; 95% CI = 95 % confidence interval.

* *p* ≤ 0.05 indicates statistical significance.

**Figure 1:**
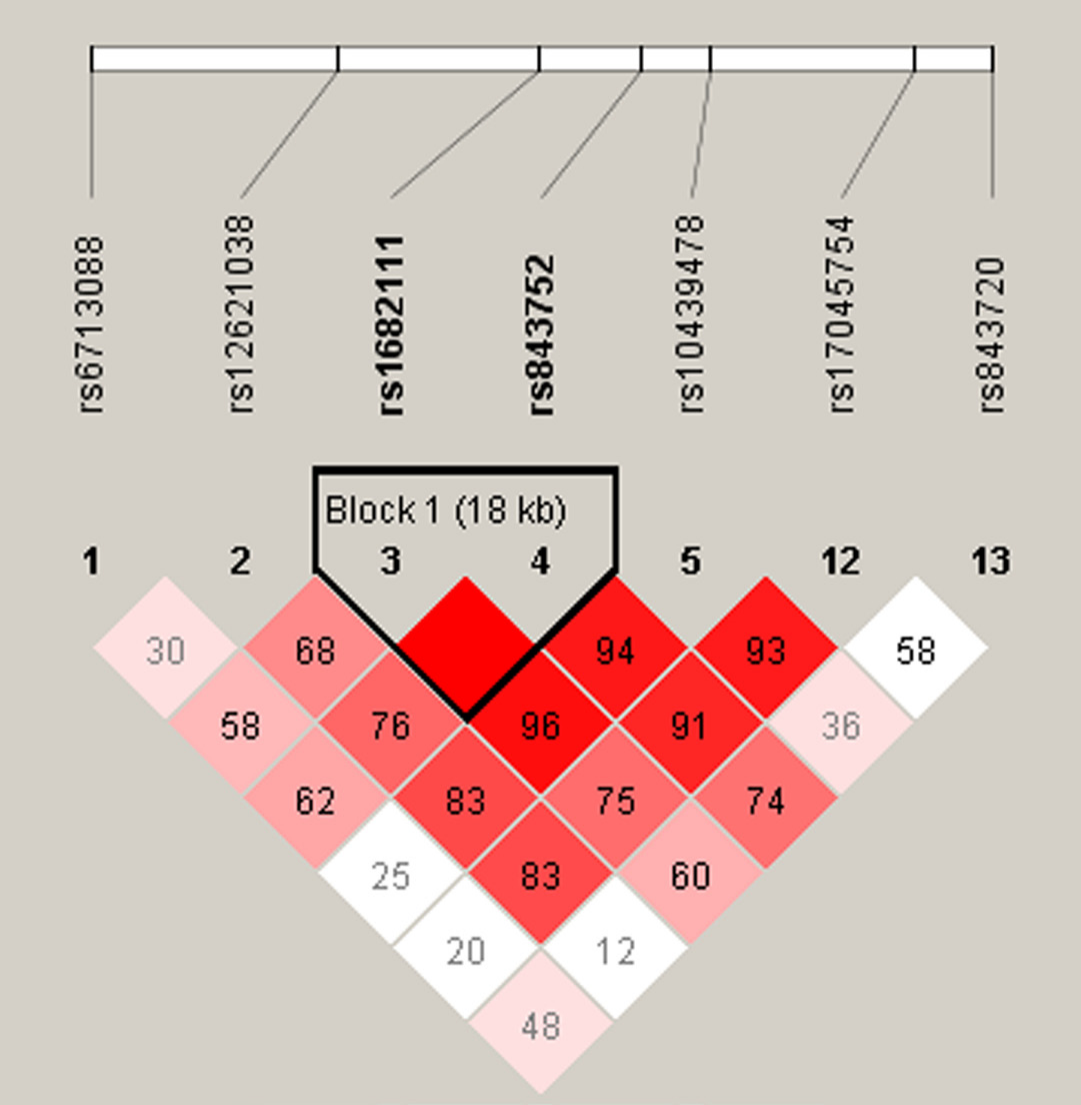
Haplotype block map for the seven SNPs analyzed in the ACYP2 gene

Wald tests were performed using an unconditional multivariate regression analysis to assess the associations between SNP haplotypes and BC risk. Interestingly, we determined that *ACYP2* haplotypes were associated with BC risk. We identified one haplotype block comprised of rs1682111 and rs843752 (Table 6, Figure 1). The AT haplotype was associated with an increased risk of BC (OR = 1.41; 95% CI = 1.01–1.95; *p* = 0.042), while the TT haplotype was associated with a decreased risk of BC (OR = 0.73; 95% CI = 0.53–0.99; *p* = 0.045) after adjustment for age, BMI, and menopause status.

**Table 6:** The associations between *ACYP2* haplotypes and breast cancer risk

Gene	SNP	Haplotype	Frequency (%)		Crude analysis				Adjusted analysis			
			Cases	Controls	OR	95% CI		*p*	OR	95% CI		*p*
*ACYP2*	rs1682111|rs843752	TG	0.30	0.30	1.02	0.74	1.39	0.921	1.01	0.73	1.39	0.953
		AT	0.32	0.26	1.40	1.01	1.93	**0.043^&^**	1.41	1.01	1.95	0.042*
		TT	0.37	0.44	0.72	0.53	0.99	**0.042^&^**	0.73	0.53	0.99	0.045*

OR = odds ratio; 95% CI = 95 % confidence interval.

& Crude Analysis.

* Adjusted for age group, BMI, and menopause status.

*p* ≤ 0.05 indicates statistical significance.

## DISCUSSION

We investigated associations between seven SNPs in the *ACYP2* gene and BC risk in a Han Chinese population. Our results indicate that rs12621038, rs17045754, and rs1682111 are associated with the risk of BC. These findings were confirmed in an analysis of two independent patient populations. Two SNPs (rs12621038 and rs17045754) were associated with a decreased risk of BC and one SNP (rs1682111) was associated with increased BC susceptibility. Finally, rs6713088 genotypes were inversely correlated with tumor size, while rs17045754 was correlated with BC recurrence.

These results deviated from the expected frequency of the T allele in the general population compared to the reported frequency of rs12621038 in the Han Chinese population. The allele frequencies reported by the 1000 Genomes Project are: C-81.4% and T-19.6%, while the HapMap project reported the following frequencies: C-54.7% and T-45.3% in a Chinese population. In different Chinese ethnic groups and in Europeans, the genotype frequencies of rs12621038 vary significantly (C-79.2% and T-20.8%). Thus, different ethnic populations exhibit different allele distributions, which can result in altered interactions with environmental effects.

AcPases are small cytosolic enzymes (11 kDa) that are broadly expressed in vertebrate tissues. They exist in two different isoenzymatic forms: the muscle and organ-common types [23, 24]. Previous studies have demonstrated that AcPase nuclease activity on DNA in an acidic environment plays a role in DNA hydrolysis during apoptosis [19, 25, 26]. AcPases also hydrolyze phosphoenzyme intermediates generated by various membrane pumps [14] and may modulate Ca^2+^ homeostasis. Calcium can regulate both cell survival and death [27]. Indeed, deregulation of Ca^2+^ homeostasis is one of the first hallmarks of apoptosis [28]. Cancer cells can evade apoptosis through decreased expression of Ca^2+^-permeable channels to prevent calcium influx, acquire resistance to prolonged endoplasmic reticulum calcium deficiency, and down-regulate mitochondrial calcium uniporters [29]. AcPase pathways may be altered in various cancers, and overexpression of *ACYP2* could decrease the risk of BC.

There are limited data on the relationship between tumor size and the risk of BC [30]. Recently, Nuyten et al. performed gene expression profiling to identify gene signatures that could predict BC [31]. We have provided the first evidence that polymorphisms in *ACYP2* are associated with clinical outcomes in BC. Our data indicate that rs6713088 is associated with tumor size and that rs17045754 may be a genetic marker that could be used to predict BC recurrence.

A limitation of this study was the relatively small dataset, which decreased the statistical power to detect effects for rare SNPs. In addition, the results of our population comparison and logistic regression analysis indicated that none of the SNPs were significantly associated with BMI, menopausal status, or age. However, some studies have demonstrated that heterogeneity between different BC patient populations and between different BC therapies can modify the association between BMI and patient outcome. While these factors may confound the associations between genetic variants and BC to some degree, the relative similarities between our case and control populations indicated any confounding effects were reduced to a minimum. No significant associations were observed between the SNPs and the risk of BC after Bonferroni correction. This may be due to our relatively small sample size, the SNP selection criteria (minor allele frequency > 5%), and inherent weaknesses of the Bonferroni correction itself (the interpretation of the results depends upon the number of comparisons performed). Multiple independent studies with large sample sizes are required to validate our findings.

## MATERIALS AND METHODS

### Patients and samples

The study participants were either newly diagnosed BC patients or cancer-free controls (based on history and screening). Participants were recruited from clinics at the First Affiliated Hospital of Xi’an Jiaotong University between August 2013 and December 2015 as part of the Disease Management Project. The inclusion criteria for the BC group were the following: age > 18 years of age and histologically confirmed BC. Patients who received chemotherapy or radiotherapy before surgery, or who had another type of cancer were excluded. Cancer-free controls were selected based on physical examinations at the same hospital and were matched with the cases according to race and age (in 5-year age groups). All participants were Han Chinese and were recruited from regions in Northwest China. Participants gave informed consent and completed a personal interview regarding risk factors, medical history, family history, and lifestyle. Blood samples were collected from all participants. The study was approved by the Department of Oncology Surgery at the First Affiliated Hospital of Xi’an Jiaotong University.

### SNP selection and genotyping

We randomly selected seven potentially function SNPs in the *ACYP2* gene (rs6713088, rs12621038, rs1682111, rs843752, rs10439478, rs17045754, and rs843720) for analysis. The SNPs were selected based on population and MAF > 5% using dbSNP (http://www.ncbi.nlm.nih.gov/projects/SNP). These SNPs represented the majority of known common variants in *ACYP2*. Genotyping was performed using a Sequenom MassARRAY RS1000 (Sequenom, Inc., San Diego, CA, USA) according to the manufacturer’s instructions. Briefly, locus-specific polymerase chain reaction (PCR) amplification was performed and the products purified by addition of shrimp alkaline phosphatase. Single base extension was then performed using primers that annealed immediately upstream of each SNP. Finally, the alleles were determined by mass spectrometry of the extended primers. The primers used for each SNP are listed in Table 7.

**Table 7:** Primers

SNP_ID	1st_PCRP	2nd_PCRP	UEP_SEQ
rs6713088	ACGTTGGATGACACA CACAGACTCCTTCAC	ACGTTGGATGGTCACC AAAACACGTAATG	gaggcCAGAATGGTCCACTAGAGA
rs12621038	ACGTTGGATGATTGT GCTAGGCACTTTAGG	ACGTTGGATGGGCA TAAGTTTTATTGCCTC	ccATTGCCTCAGCTAGACT
rs1682111	ACGTTGGATGGAATT GCTGGGTTATTTGGC	ACGTTGGATGGCCAGT GGGAATGCAAAATG	tgtcATGCAAAATGAAACAGACACTT
rs843752	ACGTTGGATGTCCTC TTTTCAGAAACCTGC	ACGTTGGATGGAGACA ACATAATGGAGGTC	cGAGTTTGGGTTTGAGGT
rs10439478	ACGTTGGATGTAGCAC AAGACCTACACTGG	ACGTTGGATGCTACAC TCTCCAGAGGAATG	TTGCTGTTTTCCCAGAA
rs17045754	ACGTTGGATGCTGTA AAAGTTCTGGCATGG	ACGTTGGATGGAAATC AGGGATATTAGTGC	caggTATTCAGCTTCCTAGAGTTA
rs843720	ACGTTGGATGCTTCAC AACACTCCTGTAAG	ACGTTGGATGAGTCAG AGCTAGACCTCTGG	ccccAATCTGTCTCAGGGTCTT

### Statistical analysis

Statistical analyses were performed using SPSS version 17.0 for Windows (SPSS, Chicago, IL, USA). Population characteristics were compared using independent t-tests (Levene’s test to assess the equality of variances) for continuous variables and Chi-square tests for categorical variables. The PLINK software package (version 1.07) was used to assess possible heterogeneous associations across populations. All populations were tested for HWE. Genotype and allele effects were analyzed using Chi-square tests. ORs and CIs were calculated from the combined results of both populations after adjustment for age, BMI, and menopause status.

## CONCLUSIONS

Our analysis indicates that rs12621038 and rs17045754 in the *ACYP2* gene are associated with a decreased risk of BC in a Han Chinese population. In contrast, rs1682111 is associated with an increased risk of BC. Given the influence of environmental factors, our results must be validated in larger cohorts, and detailed functional studies are required.
